# Isolated splenic sarcoidosis: A case report

**DOI:** 10.23938/ASSN.1131

**Published:** 2025-09-22

**Authors:** Coro Velasco Gametxogoikoetxea, Irene Fernández De Los Reyes, Fermín Jiménez Bermejo

**Affiliations:** 1 Servicio Navarro de Salud-Osasunbidea Hospital Universitario de Navarra Servicio de Cardiología Pamplona España; 2 Servicio Navarro de Salud-Osasunbidea Hospital Universitario de Navarra Servicio de Anatomía Patológica Pamplona España; 3 Servicio Navarro de Salud-Osasunbidea Hospital Universitario de Navarra Servicio de Medicina Interna Pamplona España

**Keywords:** Sarcoidosis, Spleen, Case Reports, Adolescent, Female, Sarcoidosis, Bazo, Presentación de Caso, Adolescent, Femenino

## Abstract

Isolated splenic sarcoidosis is a rare entity, but clinicians should consider it in patients with compatible clinical features. Its diagnosis is challenging due to the broad differential diagnosis, which includes hematologic and splenic neoplasms, infiltrative and inflammatory disorders, autoimmune diseases, and infections.

We report the case of a 15-year-old female diagnosed with isolated splenic sarcoidosis during hospitalization for fever of unknown origin. Histopathological examination revealed non-caseating granulomas and necrotizing granulomas. The patient showed marked clinical and radiological improvement following corticosteroid therapy, supporting the diagnosis. Despite its atypical presentation, this case highlights the importance of including sarcoidosis in the diagnostic evaluation of prolonged fever, even in young patients without respiratory symptoms.

## RESUMEN

La sarcoidosis esplénica aislada es una presentación muy poco frecuente, pero debe tenerse en cuenta en pacientes con síntomas compatibles. Constituye un reto clínico debido al amplio diagnóstico diferencial que debe considerarse, que incluye neoplasias hematológicas y esplénicas, trastornos infiltrativos, inflamatorios, autoinmunes e infecciones.

Presentamos el caso de una mujer de 15 años diagnosticada de sarcoidosis esplénica aislada durante su ingreso hospitalario por fiebre de origen desconocido. El estudio anatomopatológico reveló la presencia de un granuloma esplénico no caseificante y de granulomas necrotizantes. La respuesta clínica y radiológica observada tras el tratamiento con corticosteroides reforzó el diagnóstico. La sarcoidosis debería considerarse en el diagnóstico diferencial de fiebre prolongada, incluso en pacientes jóvenes sin sintomatología respiratoria.

## INTRODUCTION

Sarcoidosis is a systemic disease of unknown etiology, characterized by the formation of non-necrotizing epithelioid granulomas. These granulomas consist of epithelioid cells and giant cells, as well as CD4+ T cells. They are surrounded by CD8+ T lymphocytes and B lymphocytes^1^. The theory holds that sarcoidosis may result from an exaggerated immune response to a variety of antigens, leading to the proliferation and accumulation of CD4+ helper T cells^2^.

Pulmonary involvement is observed in more than 90% of sarcoidosis cases. Although extrapulmonary manifestations - both with and without lung disease - are well documented, isolated extrapulmonary sarcoidosis is uncommon, representing only about 10% of reported cases^3^^,^^4^. Isolated splenic involvement is particularly rare^4^.

We report the case of a 15-year-old female diagnosed with isolated splenic sarcoidosis during hospitalization for fever of unknown origin. This case is noteworthy for the exclusive splenic presentation of sarcoidosis, an uncommon manifestation that underscores the need for clinical awareness of rare patterns. It also emphasizes the importance of including sarcoidosis in the differential diagnosis of prolonged fever, even in young patients without respiratory symptoms.

## CLINICAL CASE

A 15-year-old girl presented to the emergency department with a two-month history of high fever (38°C), accompanied only by a cough. She had no relevant past medical history.

Initially, the fever was intermittent and associated with productive cough, pharyngeal mucus, rhinorrhea, and nasal congestion. She was prescribed two courses of antibiotics - first amoxicillin, then levofloxacin - without symptom resolution. Following the second antibiotic, she reported persisted daily fever for 19 days, peaking at 38ºC, predominantly in the evenings, and accompanied by sweating, fatigue, and a persistent cough, which had become non-productive since antibiotic use.

After two months without a definitive diagnosis, she returned to the emergency department. She reported that fever had been reasonably controlled at home by alternating ibuprofen and paracetamol. In recent days, she had developed pain in the left chest, localized between the ninth and twelfth ribs along the mid-axillary line, occurring only during coughing. She denied odynophagia, tonsillitis, airway obstruction, erythema, or pruritus. There was no history of vomiting, abdominal pain, bowel habits changes, or urinary tract symptoms. She reported no lymphadenopathy, weight loss, animal or insect contact, recent travel, or engagement in unsafe sexual practices.

On physical examination, there was tenderness in the left hypochondrium on deep palpation, without signs of peritoneal irritation or hepatosplenomegaly.

Basic blood tests showed mild anemia and leukocytosis with neutrophilia. The erythrocyte sedimentation rate (first hour) was elevated, and C-reactive protein was markedly increased at 166 mg/L [reference range: 0-5 mg/L]. All other parameters were within normal limits.

On admission, an extensive blood panel was requested, including an autoantibody profile, immunoglobulin classes and subclasses, serology and a PCR test to detect both typical and atypical microorganisms. Additional investigations included β2-microglobulin as a tumor marker, a Quantiferon test to rule out tuberculosis, and a thyroid profile. Results revealed positive antinuclear antibodies with a titer of 1:160 and a mottled fluorescence pattern. IgG antibodies against Epstein-Barr virus were positive, as was anti-EBNA (Epstein-Barr nuclear antigen) at 600 AU/mL consistent with a past infection profile. However, the Paull-Bunnell test was negative, while EBV IgM was positive. EBV PCR in the blood was negative. The remaining laboratory tests were unremarkable.

Regarding complementary tests, the chest X-ray showed no pathological signs. The COVID-19 PCR test was negative. Blood cultures were negative, as was urine sediment analysis.

Based on the above, infectious mononucleosis caused by EBV was considered as the main presumptive diagnosis. Given that the patient also reported pain in the left lower ribs, an abdominal ultrasound was performed on the third day of admission to rule out splenic complications. The ultrasound revealed a spleen of normal size with multiple hypodense lesions of irregular contours and solid content, the largest measuring 1.7 cm, suggestive of focal inflammatory pathology in the context of infectious mononucleosis.

**Figure 1:**
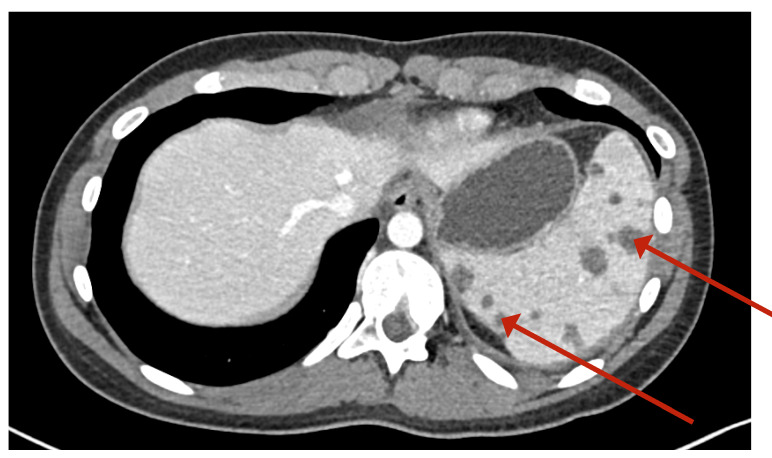
Thoracoabdominal computed tomography. Multiple hypodense nodules of indeterminate appearance are observed in the splenic parenchyma (arrows).

Due to the ultrasound findings and the persistence of fever, a thoracoabdominal CT was performed on the fifth day to evaluate for additional lesions. The CT demonstrated a spleen at the upper limit of normal size with multiple hypodense parenchymal nodules of indeterminate appearance, possibly of infectious origin (Figure 1). No supra- or infradiaphragmatic lymphadenopathy was detected.

**Figure 2:**
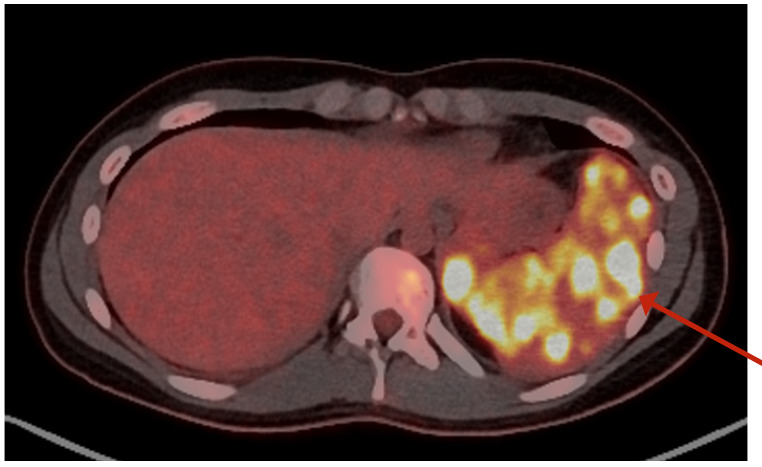
Positron emission tomography (PET). Normal spleen size with focal uptakes of elevated glucose metabolism (arrow).

After reviewing the literature, it was concluded that the hypodense splenic lesions could represent splenic abscesses in the context of EBV infection. Based on one report recommending piperacillin/tazobactam as empirical treatment^5^; this regimen was administered for seven days. However, the patient continued to experience daily fever, predominantly in the evenings, accompanied by a dry cough. Given this clinical course, a lymphoproliferative process was considered most likely, and a PET scan was requested to assess for uptake at other sites. The PET scan (Figure 2), performed on Day 10 of admission, showed a normal-sized spleen with focal uptake of high glucose metabolism. This pattern was compatible with a lymphoproliferative process, but did not exclude an inflammatory or infectious etiology.

**Figure 3:**
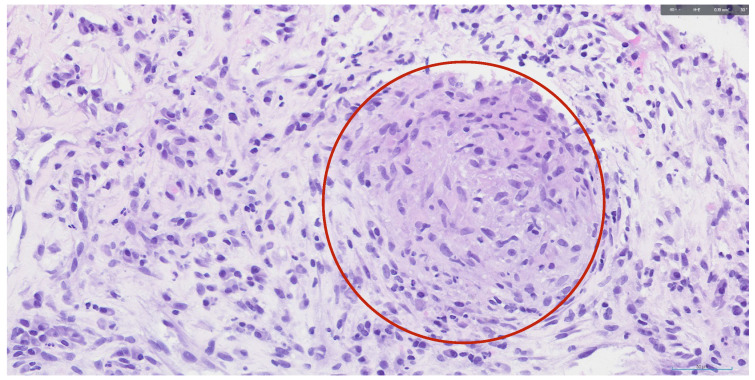
Light microscopy. Splenic non-caseating epithelioid granuloma (circle). Haematoxylin & Eosin, 400x.

In view of the need to establish a definitive diagnosis, a splenic ultrasound with biopsy was requested. Two percutaneous biopsies of one of the lesions were performed using an ultrasound-guided core needle. The samples were sent to the Microbiology Department, where no mycobacteria were detected, and to the Pathology Department. Histological examination of the splenic tissue revealed cylindrical samples containing necrotizing granulomas without specific features, as well as a non-caseating epithelioid granuloma (Figure 3). A molecular study of the splenic sample for EBV was negative and showed a polyclonal profile, with no clonal rearrangements detected in the analyzed genes.

Based on these findings, and after consultation with the Hematology Department, sarcoidosis was considered the most likely diagnosis, although marginal zone lymphoma remained a possibility.

Corticosteroid therapy was initiated with oral prednisone at a dose of 30 mg every 24 hours. The patient was discharged on Day eleven of admission. Within five days of initiating corticosteroid therapy, the patient became afebrile and asymptomatic.

A follow-up blood test two months after discharge showed mild leukocytosis with lymphocytosis due to mature lymphocytes. Angiotensin-converting enzyme 1 levels were not elevated, and the remaining laboratory parameters were within normal limits. Control abdominopelvic ultrasounds performed at two and four months after initiation of corticosteroid therapy revealed a spleen of normal size, normal morphology, and echogenicity, with no focal lesions identified. Consequently, the prednisone dose was reduced to 10 mg daily, with continued outpatient monitoring. At nine months after discharge, the patient remained afebrile and asymptomatic, allowing for gradual tapering of prednisone.

## DISCUSSION

Granulomatous inflammation of the spleen and liver is common in patients with systemic sarcoidosis^3^. However, splenic sarcoidosis in the absence of lung disease - without clinical or radio-graphic findings - is extremely rare and usually asymptomatic^3^. Some patients, however, may present with abdominal pain and systemic manifestations such as fever, as occurred in our patient, as well as malaise, weight loss, and night sweats^3^.

Kobayashi et al.^6^ published a similar case in 2021 and conducted a literature review of 11 cases of isolated splenic sarcoidosis reported up to that time. Their study suggested that splenic sarcoidosis is more prevalent in female individuals, with sweating and weight loss being the most common symptoms (5 out of 11 cases; 45%)^6^. In our updated literature review performed on July 9, 2025 using the MeSH terms “*Sarcoidosis*” and “*Spleen*” combined with the Boolean operator *AND*, we identified five new cases of isolated splenic sarcoidosis reported between late 2020 and July 2025^7^^-^^11^. We also found five additional cases reported prior to 2020^4^^,^^12^^-^^15^ that were not included in the review by Kobayashi et al.^6^.

The diagnosis of sarcoidosis is not clearly standardized^16^, and there are no universally accepted diagnostic criteria^17^. In clinical practice, the recommendations of the American Thoracic Society/European Respiratory Society/WASOG (World Association of Sarcoidosis and Other Granulomatous Disorders)^18^ are generally applied. These recommendations base the diagnosis of sarcoidosis on compatible clinical and/or radiological findings, histopathological evidence of non-caseating granulomas (though not always deemed essential), and the exclusion of other causes of granulomatous disease^16^^,^^17^^,^^19^. The wide phenotypic diversity of sarcoidosis necessitates ruling out a long list of alternative etiologies^17^. Due to the absence of pathognomonic features, sarcoidosis is always considered a diagnosis of exclusion^16^. According to the WASOG classification, the spleen-specific manifestations considered suggestive of sarcoidosis are:

- Highly probable diagnosis: biopsy findings compatible with sarcoidosis, regardless of the organ involved.
- Probable diagnosis: Low attenuation splenic nodules on CT scan, hyperattenuating splenic nodules on scintigraphy/PET scan, or splenomegaly detected by physical examination or imaging^17^.

In our patient, the presence of multiple low-attenuation splenic nodules on abdominal CT and increased uptake on PET were key imaging features consistent with the WASOG criteria for splenic involvement in sarcoidosis. Notably, there were no thoracic findings on chest CT or PET, reinforcing the isolated nature of the splenic disease.

Because sarcoid granulomas are non-specific and lack distinctive histological features, other granulomatous diseases must be excluded^3^^,^^6^^,^^20^. The most important differential diagnoses include: neoplasia (lymphoma and solid tumors), autoimmune diseases such as Wegener’s granulomatosis and primary biliary cirrhosis, drug reactions, occupational and environmental exposures^3^^,^^20^, granulomatous infections, farmer’s lung disease^3^, benign vascular tumors, metastatic cancer^2^^,^^3^^,^^20^, Langerhans cell histiocytosis^20^, multicentric Castleman’s disease, and IgG4-related disease^16^.

Cultures and stains for mycobacteria and fungi should therefore always be obtained when sarcoidosis is suspected^3^. In addition, lymphoma can mimic sarcoidosis, presenting with fever, weight loss, and lymphadenopathy^2^. A summary of the differential diagnoses considered in our case, along with the exclusion criteria applied, is presented in Table 1.

A definitive diagnosis is usually established by histological examination^6^, which may require an ultrasound-guided splenic biopsy or a laparoscopic splenectomy^21^. In our case, an ultrasound-guided core needle biopsy of the spleen was successfully performed, enabling histological confirmation without the need for splenectomy. The biopsy demonstrated non-caseating granulomas, consistent with sarcoidosis, while microbiological studies ruled out infectious agents including EBV and tuberculosis. These findings fulfilled the diagnostic requirement of excluding other granulomatous diseases.

Although non-necrotizing granulomas are the histological hallmark of sarcoidosis, necrotizing variants can occur, as in our case. This variant, known as necrotizing sarcoid granulomatosis (NSG)^1^^,^^22^^,^^23^, is rare and characterized by granulomatous inflammation, necrosis and vasculitis^3^^,^^4^^,^^6^. Clini-cal features may include subacute onset of fever, night sweats, cough, pleuritic chest pain, dyspnea, and malaise^23^ - symptoms consistent with those experienced by our patient -. NSG can complicate the differential diagnosis by mimicking tuberculosis, infarction, or lymphomatoid granulomatosis^22^. In this case, the presence of necrotizing granulomas initially raised concern for infection, but exclusion of infectious etiologies supported the diagnosis of necrotizing sarcoid granulomatosis.

Laboratory tests for sarcoidosis have limited diagnostic value^21^. The best-known marker is serum ACE concentrations, which, if elevated, may provide supportive evidence for the diagnosis. However, normal values do not rule out sarcoidosis, particularly in patients with isolated organ involvement^24^. For this reason, ACE was not initially requested in our patient.

Treatment is guided by symptoms and disease severity^3^. International consensus supports glucocorticoids as the first-line systemic treatment, with intravenous methylprednisolone pulses reserved for severe cases^25^. In our patient, treatment with oral prednisone (30 mg daily) led to rapid improvement, with resolution of fever and other symptoms within five days. Follow-up imaging after three months of treatment showed complete remission of splenic lesions, supporting the diagnosis and confirming corticosteroid responsiveness.

Exclusive splenic involvement, as observed in our patient, is extraordinarily rare. We therefore consider this case noteworthy, as the correlation between clinical findings, imaging studies, and histological documentation has seldom been reported in the literature.

In conclusion, isolated splenic sarcoidosis is an extremely rare manifestation of sarcoidosis, requiring a high index of suspicion and careful exclusion of other granulomatous diseases. Its diagnosis and management demand a multidisciplinary approach involving internists, radiologists, and pathologists. In our patient, the absence of pulmonary involvement, persistent of fever, and splenic lesions on imaging prompted extensive diagnostic evaluation. Histological confirmation via core needle biopsy was essential in excluding infectious and neoplastic causes and ultimately supported the diagnosis of necrotizing sarcoid granulomatosis. This case contributes to the limited literature on isolated splenic sarcoidosis and underscores the importance of a multidisciplinary, investigative approach to fever of unknown origin.

**Table 1:** Ruled-out diagnoses and exclusion criteria in the presented case

Diagnostic	Case-specific exclusion criteria
Active EBV infectious mononucleosis	Serology with positive IgG and anti-EBNA (past infection profile), negative EBV-PCR in blood, negative Paull-Bunnell test. The molecular study of EBV by the CISH technique in the splenic sample was negative.
Tuberculosis or infection by *Mycobacterium tuberculosis* Complex mycobacteria	The Quantiferon test was negative as was the study of *Mycobacterium tuberculosis* Complex mycobacteria in the splenic biopsy.
Parvovirus B19	Blood PCR was negative.
Citomegalovirus	Blood PCR was negative.
Atypical pneumonia	The following infections were ruled out: Q fever (*Coxiella burnetii*), mediterranean spotted fever (*Rickettsia conorii*), psittacosis / parrot fever (*Chlamydia psittaci*), toxoplasmosis, syphilis, acute herpesvirus 6 infection, acute *Chlamydia pneumoniae* infection, acute *Legionella pneumophila* infection and acute *Mycoplasma pneumoniae* infection. There was also no evidence of acute varicella-zoster virus infection, and the HIV test results were negative.
Non-specific bacterial infection	Empirical treatment with piperacillin/tazobactam showed no clinical response and the blood cultures were negative.
Splenic/marginal lymphoma or other solid tumours	Histological examination showed no atypical lymphoid proliferation or characteristic lymphocyte infiltrate, and there was an absence of supra or infra-diaphragmatic adenopathies. No clonal rearrangements were detected in the molecular study. Histological examination also showed no presence of tumour cells of other origin.
Benign vascular tumours	Hypodense lesions on CT and biopsy showing non-caseating granulomas and no vascular proliferation. The lesions disappeared after corticosteroid treatment.
Solid metastatic tumours	PET scan showing no additional uptake compared to the spleen and no neoplastic cells in the splenic biopsy.
Juvenile systemic lupus erythematosus or systemic autoimmunity	Antinuclear antibodies positive but with a low titter (1:160) and a non-specific pattern (a mottled fluorescence pattern). There were no major clinical criteria or other positive autoantibodies (negative antibodies anti-DNA and negative ENA), and complement levels (C3 and C4) were within normal limits.
Systemic vasculitis	Absence of nephropathy, purpura and ANCA negativity. No necrotising vasculitis or eosinophilic infiltration was seen in the splenic sample.
Primary biliary cirrhosis	There was no jaundice, cholestasis, pruritus or liver disorders.
Zoonosis	No exposure to animals or insects. No compatible clinical manifestations or positive serology were reported.
Castleman and IgG4-related disease	Histology not suggestive. There were no adenopathies or laboratory findings suggestive of these diseases. Serum IgG4 were not elevated
Langerhans cell histiocytosis	There were no compatible histological or radiological findings (eosinophilic granulomas are the most common). There was no bone or skin involvement.
Common variable immunodeficiency	Immunoglobulin studies (classes and subclasses) were performed as part of the protocol, but no significant findings were identified. The patient had no history of recurrent infections.
Lymphomatoid granulomatosis	There was no lung involvement, the PET/CT scan showed no lung nodules, the biopsy showed no angiocentric infiltrate, the EBV-PCR test was negative in the tissue and there was no clonality.
Occupational or environmental exposure	The adolescent patient had no history of occupational exposure or relevant environmental contact.
Drug reactions	There is no record of the patient taking drugs with the potential to induce granulomas prior to the onset of the symptoms.

ANCA: antineutrophil cytoplasmic antibody; CISH: chromogenic in situ hybridization; CT: computed tomography; EBNA: Epstein-Barr nuclear antigen; EBV: Epstein-Barr virus; ENA: extractable nuclear antigens; HIV: human immunodeficiency virus; Ig: immunoglobulin; - PCR: polymerase chain reaction.

## Data Availability

They are available upon request to the corresponding author.
